# Soft wireless sternal patch to detect systemic vasoconstriction using photoplethysmography

**DOI:** 10.1016/j.isci.2023.106184

**Published:** 2023-02-13

**Authors:** Nathan Zavanelli, Sung Hoon Lee, Matthew Guess, Woon-Hong Yeo

**Affiliations:** 1 George W. Woodruff School of Mechanical Engineering, Georgia Institute of Technology, Atlanta, GA 30024, USA; 2 IEN Center for Human-Centric Interfaces and Engineering at the Institute for Electronics and Nanotechnology, Georgia Institute of Technology, Atlanta, GA 30332, USA; 3 School of Electrical and Computer Engineering, Georgia Institute of Technology, Atlanta, GA 30332, USA; 4 Wallace H. Coulter Department of Biomedical Engineering, Georgia Tech and Emory University School of Medicine, Atlanta, GA 30332, USA; 5 Parker H. Petit Institute for Bioengineering and Biosciences, Neural Engineering Center, Institute for Materials, Institute for Robotics and Intelligent Machines, Georgia Institute of Technology, Atlanta, GA 30332, USA

**Keywords:** Health sciences, Biological sciences, Biotechnology, Bioelectronics, Engineering

## Abstract

Vasoconstriction is a crucial physiological process that serves as the body’s primary blood pressure regulation mechanism and a key marker of numerous harmful health conditions. The ability to detect vasoconstriction in real time would be crucial for detecting blood pressure, identifying sympathetic arousals, characterizing patient wellbeing, detecting sickle cell anemia attacks early, and identifying complications caused by hypertension medications. However, vasoconstriction manifests weakly in traditional photoplethysmogram (PPG) measurement locations, like the finger, toe, and ear. Here, we report a wireless, fully integrated, soft sternal patch to capture PPG signals from the sternum, an anatomical region that exhibits a robust vasoconstrictive response. With healthy controls, the device is highly capable of detecting vasoconstriction induced endogenously and exogenously. Furthermore, in overnight trials with patients with sleep apnea, the device shows a high agreement (r^2^ = 0.74) in vasoconstriction detection with a commercial system, demonstrating its potential use in portable, continuous, long-term vasoconstriction monitoring.

## Highlights

• Optimized skin-conformality enables continuous PPG monitoring from the sternum
• Improvements in sensor mechanics reduce motion artifacts compared to rigid devices
• We demonstrate high-agreement (78%) vasoconstrictions compared to WatchPAT
• Vasoconstriction monitoring could play a crucial role in monitoring cardiac disease

## Graphical abstract

**Figure:**
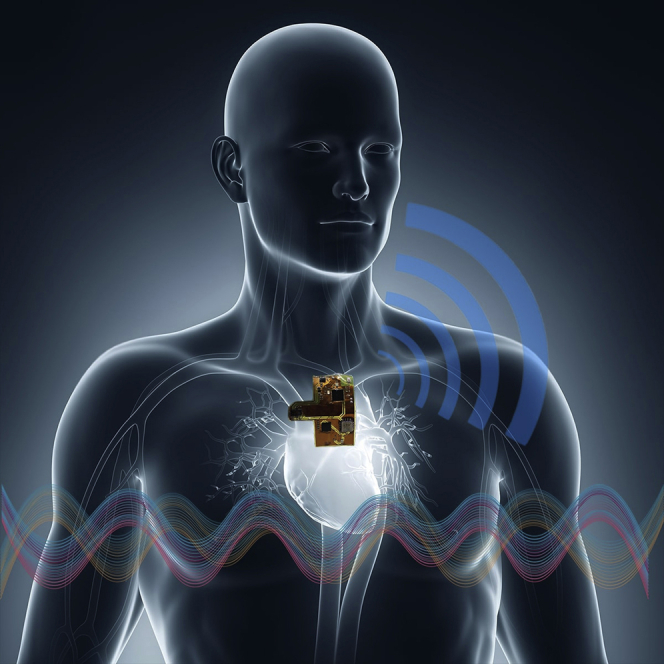

## Introduction

Vasoconstriction and vasodilation refer to the narrowing and widening of arterial pathways. This process is controlled by the concentration of calcium ions present in arterial smooth muscle cells. The body uses it as a crucial mechanism to regulate blood pressure, reduce hemorrhaging, maintain core body temperature, and partition blood flows.^1^^,^^2^ In addition to being a key player in how a healthy body maintains homeostasis, vasoconstriction is also a marker of several health conditions, like sickle cell disease, shock, migraines, asthma, glaucoma, rosacea, and chronic stress and exacerbator of several others, like Raynaud’s phenomenon, hypertension, headaches, and coronary artery disease.^3^^,^^4^^,^^5^^,^^6^^,^^7^ Furthermore, induced vasodilation is one of the most effective and well-studied means of medically reducing blood pressure, forming the basis of numerous pharmaceutical treatments.^8^ Because vasoconstriction is central to so many physiological processes, it is crucial to measure and challenging to isolate. One mechanism for vasoconstriction detection is to measure PPG signals, where an optical sensor is used to determine the pulsatile blood flow in an artery.^9^ However, PPG is rarely employed to measure vasoconstriction today because the anatomical regions where PPG is typically measured, like the fingers, ear, forehead, and foot, are perfused primarily by capillary structures, where venous pooling and respiratory modulation produce confounding signals.^10^^,^^11^ In addition, these regions are greatly affected by changes in peripheral body temperature.^11^ Instead, vasoconstriction is primarily measured with obtrusive systems, like laser Doppler flowmetry or high-pressure cuffs that eliminate venous pooling.^12^^,^^13^ Thus, a tremendous opportunity to characterize patient health and detect disease is foregone. If one could measure vasoconstriction in real time, it would be possible to assess the effectiveness and adjust the dosage of blood pressure medication, detect serious health conditions, like a sickle cell attack, before it occurs, identify long-term trends in sympathetic neural activity and patient stress, and better understand an individual’s unique response to hormonal medication.^14^

Although traditional PPG monitoring sites are incompatible with continuous vasoconstriction detection, the chest is an attractive alternative because the subcutaneous microcirculation is driven in principle by central angiosomes, which exhibit a much lesser venous pooling and respiratory effect than capillary vascularization.^15^^,^^16^^,^^17^ However, no commercial device has successfully measured vasoconstriction from the chest because the area is poorly perfused, resulting in a lower signal amplitude. The chest surface morphology is highly subject dependent and nonlinear. Thus, it is challenging to apply sufficient pressure, unlike in a finger probe, and quality skin-device contact is difficult to maintain because of motion artifacts.^10^^,^^15^ Several attempts have been made to measure PPG from the chest, but the rigid sensing materials employed have proven incapable of maintaining sufficient skin-device contact.^18^^,^^19^ Likewise, no soft sensing system has successfully implemented the necessary pressure on the PPG unit to induce high enough optical coupling between the sensor and the skin.^20^ There are a number of rigid and bulky devices that monitor vasoconstriction.^12^^,^^13^^,^^21^^,^^22^^,^^23^^,^^24^^,^^25^^,^^26^ Overall, new studies in soft material interfaces are necessary to unlock chest-based PPG’s medical potential for vasoconstriction detection.

In this work, we demonstrate a wireless, soft sternal patch with optimized skin-like electronics finely tuned to measure vasoconstriction from the sternum continuously. Analytical, computational, and empirical studies in soft materials and mechanics yielded a pressure application and elastomer-dampening system capable of mitigating motion artifacts in the PPG signal from the chest and producing a highly conformal skin-device contact. First, a human subject study determined that the mid-sternum is the optical chest location for vasoconstriction detection, and the final device optimization was tuned for this region. Next, vasoconstriction detection was validated during controlled breath holds and core body temperature modulation in healthy subjects, demonstrating high detection efficacy across all subjects. Finally, in overnight trials, vasoconstriction induced by sympathetic arousals in patients with sleep apnea was detected, and the device demonstrated high agreement with the FDA-approved, commercial WatchPAT system. The fundamental studies in chest-based PPG monitoring are valuable for their immediate medical value and general applicability to the study of soft sensors and mechanics. Furthermore, the results reported here are of general interest for PPG recording in a wide range of traditionally unfavorable anatomical regions.

## Results

### Device design and functions

**Figure 1:**
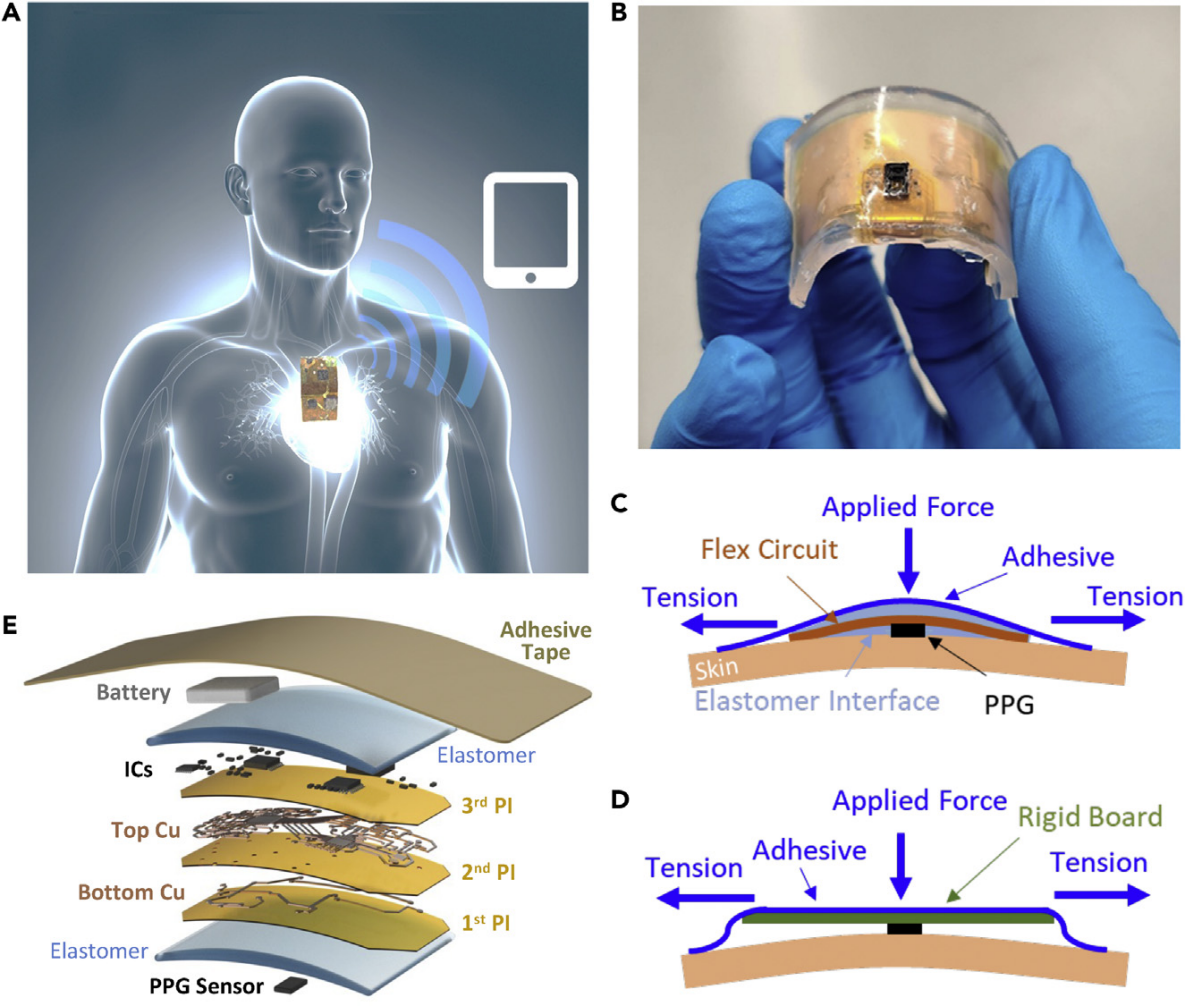
Overview of a soft sternal patch (A) Illustration of the soft sternal patch that can interact with an external device for wireless data recording. (B) Image of the soft patch showing flexibility. (C and D) Illustrations showing a soft device’s skin-contact quality (C) compared to a rigid device case (D) that cannot adhere to the skin and is susceptible to rocking and translational motion artifacts. (E) Details of the soft sternal patch’s components, including multiple layers of a sensor, polymers, metals, electronic chips, and a battery on a soft adhesive tape.

In this work, we developed a soft sternal patch comprised of a photolithographically patterned, ultrathin flexible circuit embedded in an elastomeric membrane and functionalized with integrated components. The system adheres to the chest in a minimally obtrusive manner through the natural van der Waals forces exhibited at the skin-elastomer interface and through a biocompatible tape contacting the skin at the device periphery. Figure 1A illustrates the medical goal of this research: a simple, imperceptible patch mounted on the chest to measure PPG and communicate wirelessly with a receiver tablet. A detailed description of the fabrication process of the multi-layered device appears in Figure S1. An image of the finalized device with extruding PPG unit during bending is shown in Figure 1B. Measuring PPG from the chest is a significant challenge because the basal microcirculation in this region is poorly perfused; the anatomical complexity of the chest presents difficulties in device conformality. The lack of a readily available pressure application mechanism, like those present at the finger and ear, complicates the incorporation of sufficient downward force on the sensor. To achieve this, we hypothesize that a soft system where a PPG unit is compressed into the skin by a uniaxial tape applying pressure through dampening, skin-like elastomer, and highly flexible board can produce a sufficient skin-device contact to measure excellent quality sternal PPG. Details of the circuit design appear in Figures S2 and S3.

Figure 1C illustrates the hypothesized pressure application mechanism. As shown in the illustration, the system was hypothesized to compress around the PPG unit, creating a highly conformal contact. The downward pressure on the PPG unit can be modulated by the offset height of the unit relative to the board and the tensile stress in the tape. A flexible tape was chosen as the pressure application tape because it rapidly increases stiffness at a specific tension, allowing simple instructions to produce consistently applied pressures across subjects. This was validated by having all overnight test subjects apply the devices independently in their own homes without researchers present or external application assessment. This mechanism is contrasted with Figure 1D, which illustrates a rigid system that clearly is incompatible with a quality skin-sensor contact and highly susceptible to motion artifacts. This conceptual stack-up is realized in the final circuit through the layers illustrated in Figure 1E.

**Figure 2:**
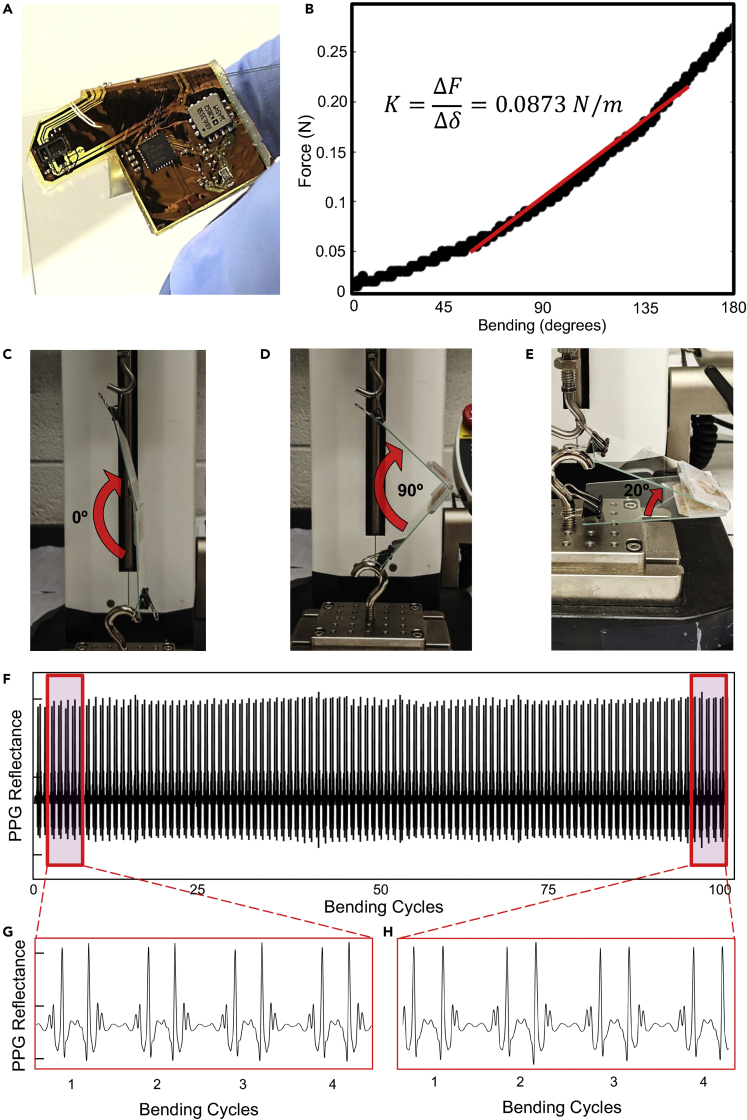
Experimental results for device mechanics and reliability (A) Image of the flexible device folded on a glass slide. (B) Calculation of the device bending stiffness (in terms of displacement of the tip during bending). (C−E) Experimental setup for a bending test showing the device with (C) 0°, (D) 90°, and (E) 160° bending, respectively. (F) PPG signals recorded throughout 100 rounds of cyclic bending. The signals are comprised of light from the ambient environment. (G and H) Signal waveforms at the (G) beginning and (H) end of the trial, demonstrating no deterioration.

Mechanical studies were then conducted to empirically determine the soft device’s bending stiffness and reliability. First, the device was bent through 180⁰ over a 3 cm radius via a Mark-10 ESM303 Advanced Motorized Test Stand. An image of the device seamlessly bending over this radius is shown in Figure 2A. The stiffness was calculated as a function of the board’s tip displacement and the resultant stiffness of <0.1 N/m indicates exceptional flexibility (Figure 2B). Second, the device was assessed in bending to 160⁰ over the same radius for 100 cycles. The reduced angle was chosen due to limitations in the testing setup, not an assumption about the device’s reliability. Images of the test setup are shown in Figures 2C–2E. During the 100 bending cycles, PPG signals were wirelessly transmitted via Bluetooth to an Android receiver. As shown in Figure 2F, the signals maintained excellent quality throughout the recording. Sample signals at the beginning (Figure 2G) and end (Figure 2H) of the trial indicate no difference in electrical functionality. Next, finite element analysis (FEA) was conducted to simulate the board’s compression into the skin to verify that the proposed elastomer dampening system yields a fully conformal contact. When a linear displacement is applied, the simulated board seamlessly conforms to the skin when a skin stress of only 500 kPa is achieved, which is far below the range necessary for vessel occlusion or pain and easily achieved with force applied by a tensed tape.^27^ Results of this FEA experiment are shown in Figure S4 and compared to a simulation of a traditional polyimide flex PCB that cannot conform to the skin at the same applied stress.

Finally, the device was designed specifically to reduce motion artifacts in sternal PPG, which is the main limiting factor preventing traditional rigid systems from utilizing this anatomical region. Here, we assume that non-conformal motion of the board will primarily be caused when the skin bends, stretches, translates laterally, undergoes torsion, or experiences surface waves. However, stretching is likely to dominate, especially during respiration. In this model, the highly flexible board is well suited to handle concave bending and stretching and maintain conformality because of the unique elastomeric dampening design. When the skin stretches, the tension in the tape increases, causing the pressure applied to increase, further pushing the board into conformal contact with the skin. When the user’s skin folds inwards, like when a user hugs, for instance, the highly flexible circuit can seamlessly bend with the skin, and the dampening in the elastomer prevents the applied PPG pressure from becoming excessive. Because the tape is uniaxially stretchable, it easily allows for breathing motion in the horizontal plane while limiting torsion, although the circuit’s flexibility could likewise handle skin torsion. Before conducting trials in human subjects, the device was tested for biocompatibility by being worn for 3 consecutive days and observing whether any skin irritation occurred. The results are shown in Figure S5, which shows that only very minor skin reddening was noted, even after 3 days of continuous wear. This indicates a high degree of biocompatibility.

### Device functionality and optimization study of device locations

**Figure 3:**
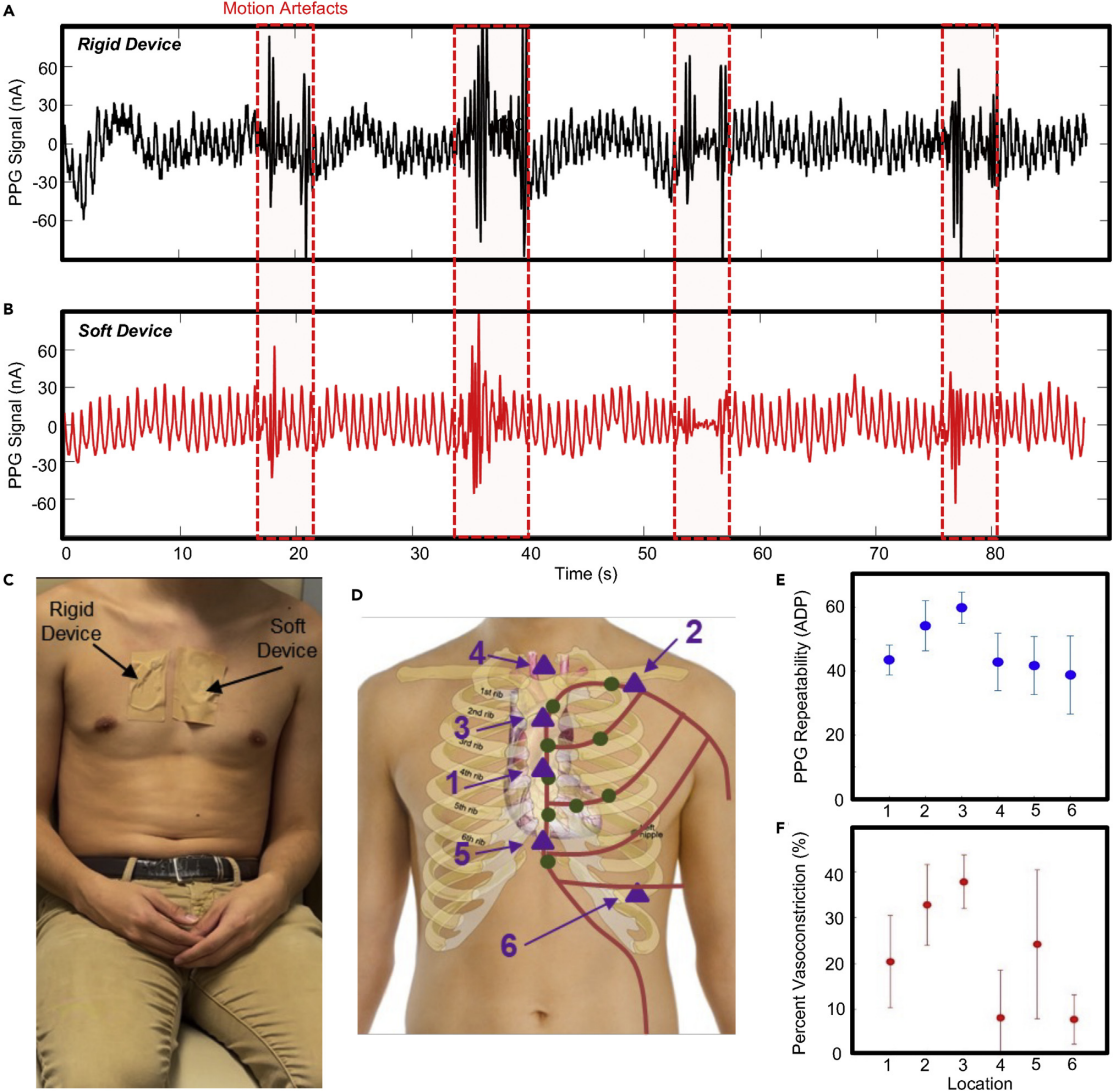
Device functionality and location optimization studies (A and B) PPG signals during rest and perturbations of the skin induced by stretching and pulling for both the (A) rigid and (B) soft devices, respectively (see Video S1). (C) Image of the experimental setup. (D) Illustration of the measurement sites tested (triangles) with major vessels (red line) and major angiosomes (green). (E and F) Charts of (E) PPG signal repeatability and (F) percent vasoconstriction during breath hold tests as a function of location (5 subjects, 3 trials, error bars of 1 SD).

The first human-subjects trial was conducted to assess the hypothesis that the soft mechanics innovations introduced here can improve signal quality and reduce motion artifacts compared to a rigid device. As shown in Video S1, signals were simultaneously recorded in both a soft device and a rigid alternative with identical components and footprint during skin perturbations. This video clearly shows that the magnitude and duration of motion artifacts is increased in the rigid device compared to the soft device. These results are further shown in Figures 3A and 3B, which show comparison unfiltered PPG data between the rigid and soft devices, respectively. The experimental setup is also shown in Figure 3C. After the optimal device mechanics were determined, the sensing performance was validated with human subjects, and the optimal anatomical region on the chest was identified. Multiple subjects performed three rounds of controlled breath holds of 30 s in duration while wearing the soft device on six different sternal regions, as shown in Figure 3D. In a real-life clinical application, it is unlikely that the device placement could be precisely guaranteed; thus, the soft device was placed solely based on visual inspection with the aid of the illustration in Figure 3D. In this illustration, the major vessels are shown in red, and areas of high angiosome density are shown in green. For each trial, the PPG signals were assessed for repeatability and percent vasoconstriction (Figure S6). The PPG repeatability is defined by the ratio of the signal autocorrelation at zero delay to the average of the autocorrelation at delays of one, two, and three beats. This metric captures how similar a delayed copy of the signal is to the original, thus providing a surrogate measure for signal-to-noise ratio for complicated signals. The process is summarized in Figure S7. The percent vasoconstriction is defined as the local minima divided by the 60 s running average of the signal’s Hilbert analytic envelope. The envelope calculation is summarized in Figure S8 , and it captures the magnitude of the signal over time. Here, this Hilbert analytic envelope magnitude is used to determine the subcutaneous arterial perfusion in the skin, a contraction of which is considered a vasoconstriction. The overall processing flow is summarized in Figure S9. Note that the periodicity is used to reject high noise beats from assessment in the determination of vasoconstriction. Across all sites, an acceptable PPG repeatability was demonstrated as the subject rested in a supine position. A sample ensemble of PPG waves during a 2-min recording from location 3 is shown in Figure S6, clearly indicating a high ability to detect crucial PPG waveform parameters and timing intervals. The overall repeatability results are shown in Figure 3E, demonstrating that location 3 had the highest repeatability. A Z-test between location 2 and 3 shows that the two distributions are statistically different with a *Z* score of 4.21. The average vasoconstriction magnitude, reported in percent, is captured in Figure 3F. Here, the vasoconstriction distributions for location 2 and 3 show marginal statistical difference, with a *Z* Score of 2.41 corresponding to a p value of 0.082, for the hypothesis that both datasets are derived from the same statistical distribution. Based on the increased repeatability and marginally increased vasoconstriction, location 3 was selected as the optimal device placement, although location 2 was also deemed acceptable. However, location 2 is more susceptible to motion artifacts due to the curved nature of the collarbone, and the device would be visible under most garments. Therefore, location 3 is deemed the most appropriate measurement location for continuous vasoconstriction detection.

Video S1. Study of sternal PPG motion artefact reduction in the soft device compared with a rigid alternative

### Vasoconstrictions from controlled studies

**Figure 4:**
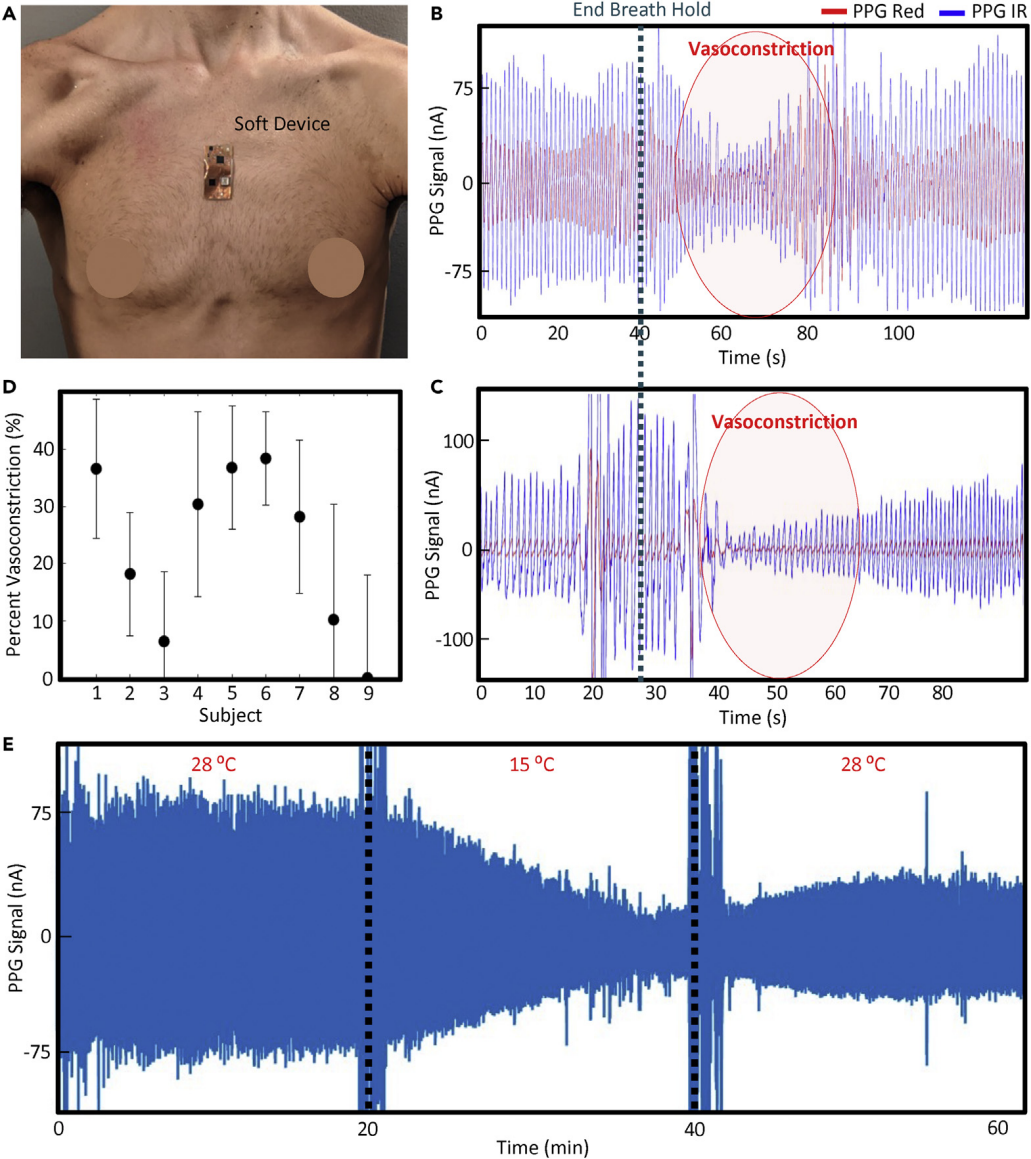
Vasoconstrictions in controlled experiments (A) Image of the soft device on a subject’s chest. (B and C) Representative vasoconstrictions observed upon the termination of breath hold. (D) Summary statistics of percent vasoconstriction during breath hold experiments across subjects (5 trials, error bars of 1 SD). (E) PPG signals from the soft device during changes in ambient temperature, demonstrating a strong ability to detect exogenously induced vasoconstrictions.

After the device placement was optimized, additional controlled experiments in human subjects were conducted to validate the system’s efficacy in endogenous and exogenous vasoconstriction detection. A breath-hold may induce vasoconstriction when chemoreceptors identify a decrease in oxygen saturation, but this response can vary greatly depending on a subject’s unique physiology. Figure 4A shows the device placed on a subject’s sternum during one simple breath hold test where subjects held their breath for 30 s, and Figures 4B and 4C show representative vasoconstrictions from two different subjects. In these tests, both the red and infrared (IR) PPG waveforms are collected. Because the penetration distance of light into the skin increases with wavelength based on the Beer-Lambert Law, this allows us to report both the dermis (red) and hypodermis (IR) perfusion. As shown in the figures, the vasoconstrictions manifest differently in each subject. In Figure 4B, the constriction is characterized by a rapid onset and a rapid termination. In Figure 4C, the constriction is rapid, but the recovery is elongated. The constrictions also manifest similarly in both the red and IR signals. However, the IR signal has notably higher amplitude and signal repeatability (increased repeatability by 41% SNR on average across 9 subjects). Thus, the IR signal was used for all further analysis. Regarding the differences in vasoconstriction morphologies, we hypothesize that each subject will react differently to the breath hold based on their individual physiologies. In both cases, the soft patch can accurately capture the subject’s vasoconstriction. Motion artifacts occurred during the termination of breath hold, which is to be expected due to the aggressive chest motion. Still, the device immediately regained excellent conformality and recorded unperturbed PPG waveforms.

The vasoconstriction results for all 9 subjects across 5 trials are summarized in Figure 4D, which shows that the device is capable of detecting vasoconstrictions across a wide range of vasoconstriction percentages and subject variabilities. Interestingly, varying percent blood flow constrictions were observed both between subjects and for the same subject across different trials. Given that there is no clinical comparison here, an additional study was conducted to validate our ability to differentiate between small, medium, and large constrictions, which we discuss in the next section. We may conclude here, however, that the device is capable of a binary distinction between events and no events, as vasoconstrictions were observed in all 5 trials for 5 subjects and 91% of trials overall (41/45) in all 9 subjects. Next, we validate the sensor’s ability to detect exogenous vasoconstrictions by considering the case of changes in core body temperature. A subject was instructed to remain outdoors at a 28°C ambient temperature for 20 min before entering a room at 15°C. The subject then returned outdoors, allowing for an assessment of constriction and dilation due to ambient temperature changes. The results of this experiment are shown in Figure 4E, where the constriction and dilation are clearly shown. Thus, we demonstrate a high ability to detect endogenous and exogenous vasoconstrictions in healthy control subjects.

### Assessment of vasoconstrictions in subjects with sleep apnea

**Figure 5:**
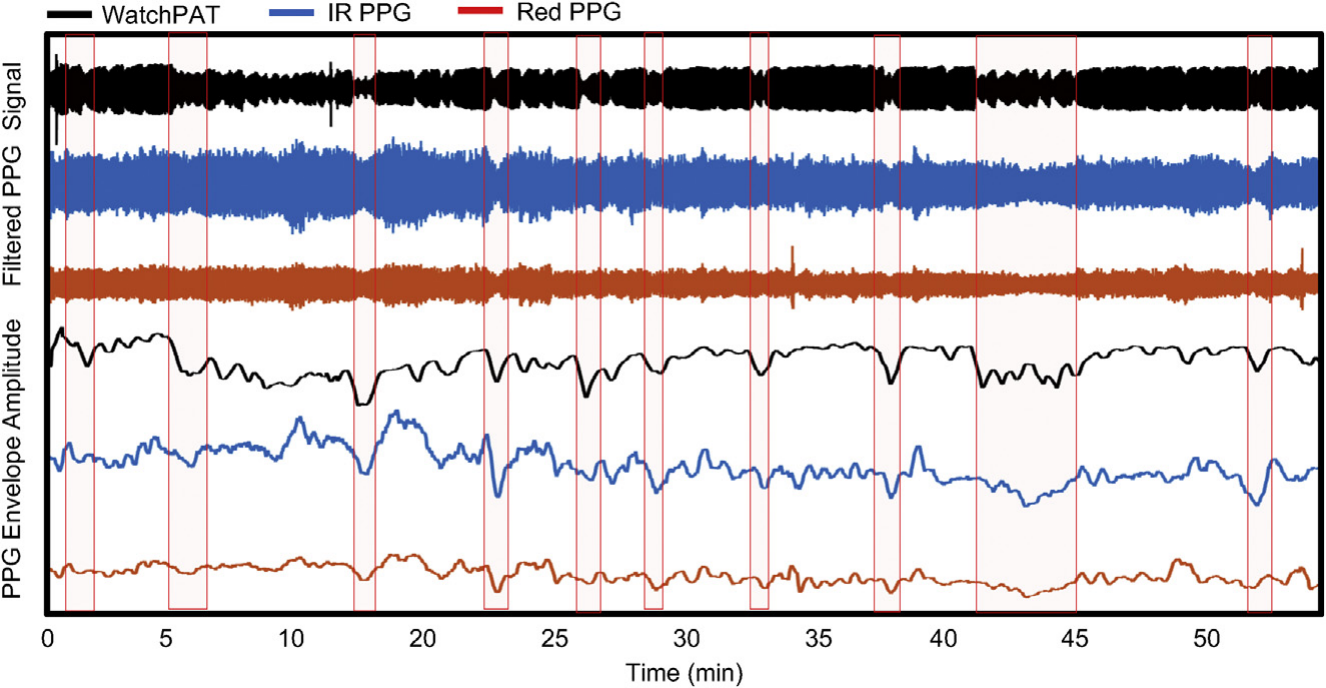
Vasoconstriction in overnight trials with patients with symptomatic apnea Data showing an overnight recording of the IR and Red PPG signals compared to a simultaneous recording of WatchPAT’s PAT signals with annotated apnea events.

Finally, the soft device’s ability to detect naturally occurring vasoconstrictions in patients with sleep apnea resulting from sympathetic arousals during sleep was studied. 8 nights of sleep were recorded across six subjects. The subject who exhibited the highest physiological propensity for vasoconstriction, as determined by their average vasoconstriction magnitude on the first night, was selected to perform a second night with a clinical grade comparison device, the WatchPAT probe. Figure 5 shows a representative hour of sleep showing the WatchPAT signal compared to the soft device’s PPG signal. The filtered waveforms correspond to the upper three signals, and the Hilbert envelope the lower, respectively. Here, each of the apnea events is automatically classified based on WatchPAT’s proprietary algorithm, which also considers heart rate, respiration rate, and actigraphy. In Figure 5, the times corresponding to each identified event are highlighted in red. As shown in the figure, there is a high agreement between vasoconstrictions in the WatchPAT signal and both the red and IR PPG signals from the soft device. However, the agreement is not perfect, both with respect to the constriction magnitude and presence of constriction. Given that there is no clinical comparison device that measures vasoconstriction from the chest, this result is potentially due to variances in physiological response based on anatomical location. Despite any such differences, the soft device and WatchPAT probe do demonstrate a clear correlation.

**Figure 6:**
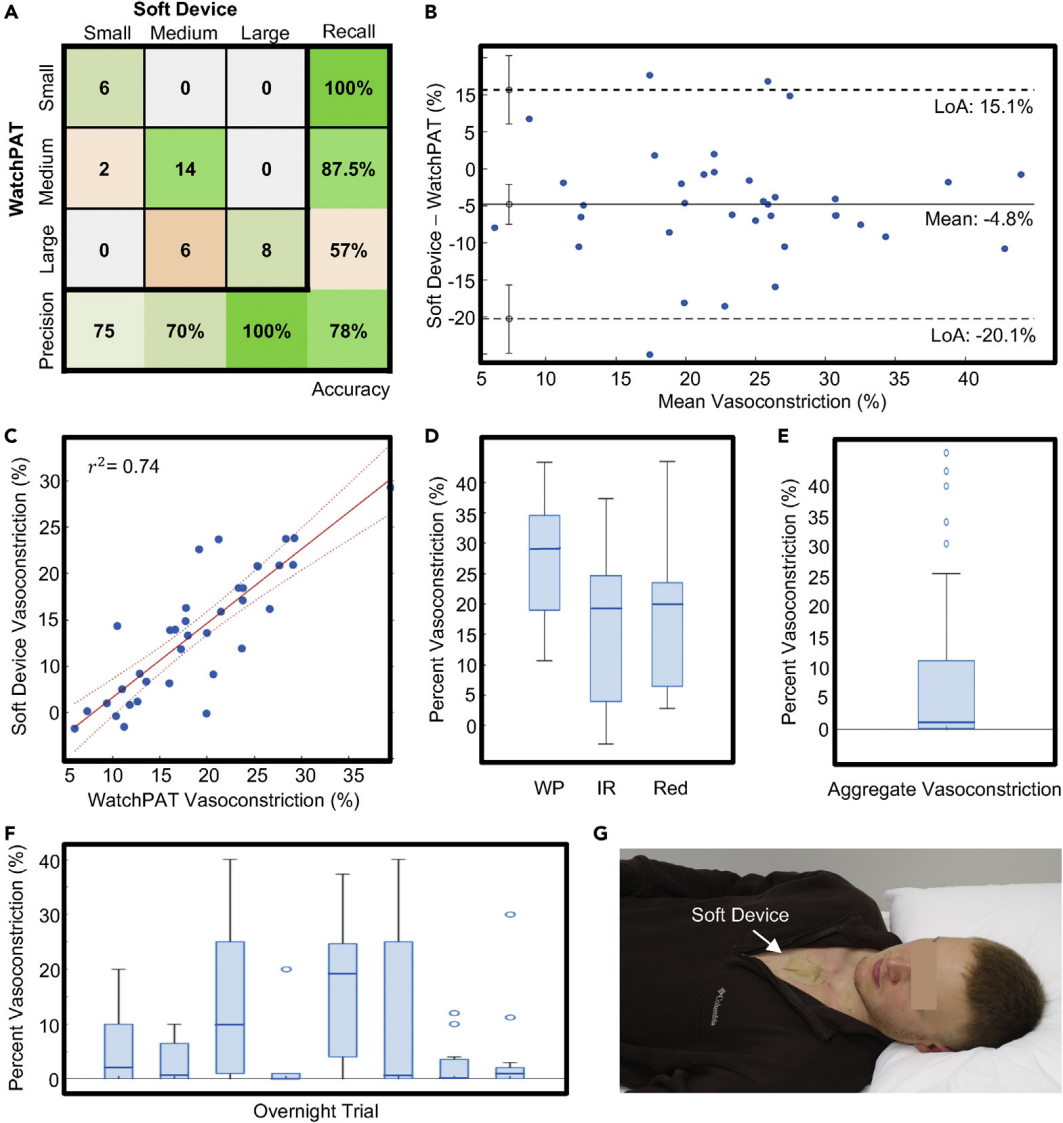
Quantitative assessment of apnea-induced vasoconstrictions (A) Confusion matrix relating the identification of small (0%–15%), medium (15%–30%), and large (30%–50%) vasoconstrictions in the comparison of soft device and WatchPAT. (B) Bland-Altman Diagram for the data reported in (A) (n = 36, error bars of 1 SD). (C) Correlation plot for the data in (A) (n = 36). (D) Percent vasoconstriction for eight overnight trials (n = 36, box 25–75 percentiles, whiskers extreme values). (E) Percent vasoconstriction aggregated to include all eight nights, showing a non-zero vasoconstriction (n = 231, box 25–75 percentiles, whiskers extreme values). (F) Boxchart comparison of vasoconstriction magnitude across all events. with P-value < 1e-6. (n varies from 13–41, box 25–75 percentiles, whiskers extreme values). (G) Image of the soft device on a sleeping subject.

This correlation is quantified in Figure 6A, which shows a comparison of vasoconstrictions in both the soft device and WatchPAT comparison when discretized into small (0%–15%), medium (15%–30%), and large (30%–50%) bins. Here, the soft device demonstrates a 78% agreement with the WatchPAT comparison in identifying systemic vasoconstrictions, according to the above schema. The specific nature of each missed event provides further support for the soft device’s detection efficacy. As Figure 6A demonstrates, all the missed events occurred because WatchPAT detected a larger vasoconstriction than the soft device. The statistical relationship between the two datasets is further investigated in the Bland-Altman plot in Figure 6B and the correlation plot in Figure 6C, respectively. In Figure 6B, the mean is shown to skew toward a higher observed vasoconstriction in the WatchPAT device than the soft device, but the variance remains consistent as the mean increases. The limits of agreement are summarized in the figure. Next, the correlation plot in Figure 6C shows a highly linear relationship between the two device’s simultaneous vasoconstriction recordings across all 36 events, resulting in an r^2^ of 0.74. Furthermore, Figure 6D shows the distribution of all events for both the soft device and the red and IR channels of the soft device (the blue line indicates average), and it demonstrates that the WatchPAT device, whether due to physiological differences or variations in applied pressure, has a higher average estimate. Therefore, it is highly likely that the remaining 22% inaccuracy is due to this systemic bias rather than a lack of detection efficacy. This result compares very favorably with alternative wearable vasoconstriction monitoring approaches, as summarized in Table 1. In addition to this study, data were collected in seven additional nights, and the overall vasoconstriction response is summarized in Figure 6E, and the individual responses are shown in Figure 6F. Although each patient had a varying degree of vasoconstrictive response, the soft device is capable of detecting constrictions in all subjects. An image of the soft device placement on a subject is shown in Figure 6G.

**Table 1:** Comparison of devices for systemic vasoconstriction monitoring

Reference	Form Factor	Continuous Use	User Comfort	Modality	Location	Validation
This work	Soft sternal patch	Yes	Great – soft and breathable	PPG	Chest	78% accuracy compared to WatchPAT
WatchPAT (26)	Rigid finger probe	No	Low – strong applied pressure	PPG	Finger	R = 0.76 for apnea detection compared to PSG.
21	Camera system	No	Low – user must remain still	Image PPG	Whole Body	Qualitative perfusion comparison to intestinal tissue.
22	Rigid device	Motion artifacts	Medium – like a hearing aid	PPG	Inner Ear	SpO2 validated. Vasoconstriction detection suggested, but not validated.
23	Rigid Patch	Motion artifacts	Medium – device’s rigidity	PPG	Chest	SpO2 validated. Vasoconstriction detection suggested, but not validated.
24	Rigid Clip	No	Low – clip on finger	PPG	Finger	Linear correlation between measured perfusion and quinine in a mouse model.
12	Pressure Cuff	No	Low – strong applied pressure	PPG	Calf	Linear correlation between measured perfusion and norepinephrine concentration in a pig model.
13	LDF Probe	No	Low – requires in hospital setting	Laser Doppler Flowmetry (LDF)	Whole body	0.58–0.88 interclass correlation between LDF and laser speckle contrast imaging up to 35% constriction.
25	LDF Probe	No	Low – requires in hospital setting	LDF	Whole body	VP1-HP LDF probe saw a 15.5% increase in vasoconstriction magnitude compared to a VP12 probe.

## Discussion

We report a soft wireless wearable patch with mechanics optimized to record vasoconstrictions in PPG signals from the chest. This work includes computational, empirical, and human pilot studies to design a skin-sensor interface based on a compressed elastomer and highly flexible microfabricated system. This system was used to identify the optimal measurement location on the chest, and then finalized based on the unique anatomical characteristics of the sternum. The mechanical insights derived from these studies were validated in controlled experiments consisting of endogenous and exogenous vasoconstrictions, where the device proved highly capable of identifying vasoconstrictions. Finally, the device was tested in symptomatic patients to determine if vasoconstrictions caused by obstructive sleep apnea could be detected. In a simultaneous study with the commercial gold-standard method using WatchPAT, an accuracy of 78% was reported, primarily due to a magnitude bias, not noise. Furthermore, the device demonstrated a high ability to detect vasoconstrictions across various magnitudes. As validated in the preceding studies, the mechanical insights presented here enable a new method for continuously detecting vasoconstrictions in real time, which has significant medical applications. For instance, this device could be used to identify the degree of vasoconstriction induced by blood pressure medication in a small trial dose, and the medicine’s physiological impact could be tracked over time. This would allow for precise medicine dosing, avoiding the risk of serious side effects. In addition, this device could be implemented to detect the warning signs of a sickle cell crisis, monitor shock victims, and assess sympathetic nervous system activation in patients with sleep disorders. Furthermore, the simple sternal patch could serve as an initial screening, augment, or even replace expensive and low-throughput MRI and CT scan to assess contracted vessels. Overall, the mechanical insights and integrated electronics presented here enable a new paradigm of continuous vasoconstriction monitoring with potentially significant medical applications. In addition, the fundamental knowledge gained from this work is broadly applicable to monitoring bio-signals from non-traditional anatomical regions, like the sternum.

### Limitations of the study

One limitation of this study is the small sample size used to assess the device’s mechanical coupling with the skin. As shown in Tables S1−S3, subjects with a variety of ethnicities, body types, and skin tones were selected, but it remains unclear if the results from this small subset are indicative of the broader human population. Furthermore, how individual physiological differences will affect the degree of vasoconstriction manifest on the chest under various medical conditions remain to be seen. Therefore, we anticipate conducting additional translational studies and medical collaborations to identify this device’s medical and diagnostic utility. Another limitation involves the photolithographic patterning used to fabricate the circuit. Our future study will develop screen printing and inkjet printing methods to fabricate flexible circuits, upon which we elaborate in the two reviews.^28^^,^^29^

## STAR★Methods

### Key resources table

**Table:** 

REAGENT or RESOURCE	SOURCE	IDENTIFIER
**Chemicals, peptides, and recombinant proteins**		

Eco-Flex 00-30	Smooth-On	00–30
PDMS elastomer	Sylgard	184
PDMS curing agent	Sylgard	N/A
Easy Release 2831	Mann	2831
Acetone 99.5+%	Alfa Aesar	99.5+%
Isopropyl Alcohol	Lab Chem	Isopropyl Alcohol
PI2610	HD microsystems	2610
Copper	Sigma Aldrich	326445
Solder Paste	Digikey	SMDLTLFP10T5-ND

**Deposited data**		

Device Firmware	https://github.gatech.edu/nzavanelli3/soft_sternal_patch/tree/master/embedded_firmware/pca10040/s132	N/A
Device Android App	https://github.gatech.edu/nzavanelli3/soft_sternal_patch/tree/master/android/Android-ADXL355-SPO2-monitoring-sync-v3	N/A
Signal Processing and Machine Learning	https://github.gatech.edu/nzavanelli3/soft_sternal_patch/tree/master/signal_processing/Matlab_v2	N/A

**Software and algorithms**		

Device Firmware	https://github.gatech.edu/nzavanelli3/soft_sternal_patch/tree/master/embedded_firmware/pca10040/s132	N/A
Device Android App	https://github.gatech.edu/nzavanelli3/soft_sternal_patch/tree/master/android/Android-ADXL355-SPO2-monitoring-sync-v3	N/A
Signal Processing and Machine Learning	https://github.gatech.edu/nzavanelli3/soft_sternal_patch/tree/master/signal_processing/Matlab_v2	N/A

### Resource availability

#### Lead contact

Further information and requests for resources and reagents should be directed to and will be fulfilled by the lead contact, Woon-Hong Yeo (whyeo@gatech.edu).

#### Material availability

All materials used in this study may be readily purchased from the suppliers listed in the key materials table.

### Experimental model and subject details

All human subjects details are provided in Tables S1−S3, respectively.

### Method details

#### Device fabrication

The soft patch was microfabricated using photolithographic patterning on a polydimethylsiloxane–coated Si wafer and two-stage transfer to an Ecoflex 00-30 elastomer, as described in section S1 and Figure S1 and demonstrated previously [27,28]. The circuit comprises top and bottom copper layers connected with etched vias and three polyimide dielectric layers. The integrated circuit components are soldered on top of pads exposed in the top copper layer.

#### Device reuse

The soft patch can be reused multiple times after undergoing a basic cleaning and an application of a new bottom adhesive layer. First, the tape is removed, and the device is sprayed with isopropyl alcohol. The device is then wiped clean with a wipe. Then it is wiped with ethyl alcohol. Finally, a thin layer of silbione is applied to the bottom of the device to form an adhesive layer with the skin.

#### Circuit information

The circuit (Figures S2 and S3) is equipped with a 150 mAh LiPo battery operating at 3.7V and downregulated to 3.3V by a TPS63001 voltage regulator and 1.8 V by a TPS62746 voltage regulator, respectively. The 3.3V is used solely to power the PPG sensor (MAX30102) LEDs. All other components operate on 1.8V. Battery life exceeds 10 h on a single charge with a power draw of <15 mA. The battery is recharged with a magnetic connector prior to each study. Central processing is handled by an nRF52832 BLE SoC, which communicates via I2C with the MAX30102 PPG sensor.

#### Experimental study for circuit bending

The circuit was progressively bent by a MARK-10 ESM303 motorized force measurement stand. The device was placed on two glass slides and bent down the interface between the slides. Device electrical performance was ensured by assessing Bluetooth transmission of PPG signals.

#### Finite element analysis for PPG pressure optimization

Finite element analysis (FEA) was conducted to simulate the conformal contact of the PPG unit to the skin for both a soft and rigid board (Figure S4). The skin consisted of a 100-μm-thick epidermis layer and a 2-mm-thick dermis layer. A PPG unit was partially embedded into a 2-mm-thick elastomer layer and polyimide layer for the soft and rigid boards, respectively. The contact conditions were set between the PPG component and the simulated skin, and a linearly applied displacement was introduced on the top surface while the bottom was fixed. The elastomer and skin layers were meshed using C3D8R elements. The following material properties of Young’s modulus (E) and Poisson’s ratio (ν) were used for the model: E_elastomer_ = 1 MPa and ν_elastomer_ = 0.49; E_pi_ = 4.0 GPa and v_pi_ = 0.34; E_epidermis_ = 1 MPa and ν_epidermis_ = 0.48; E_dermis_ = 0.2 MPa and ν_dermis_ = 0.48.

#### Sensing components

PPG is sensed through the MAX30102 sensor. Raw PPG waveforms are sampled at 200 Hz with a proprietary filter. This signal is oversampled by a factor of 4, resulting in a 50 Hz digital signal. Analog to digital conversion is handled by circuitry internal to the MAX30102, and the digital signal is 18 bits. In addition, the MAX30102 incorporates proprietary ambient light cancellation through track/hold circuitry.

#### Wireless communication

PPG data is broadcast from the nRF52832 BLE SoC in a 240-byte buffer following traditional GATT protocol. The Android tablet receives these BLE transmissions from the soft sternal patch device via a custom-designed GATT client app. Data are converted from binary to double, assigned a timestamp, and automatically saved in a CSV file in real time on the Android device. Data are plotted in real time so that the user can verify function from the tablet. The data is saved in a CSV file, which may be exported from the android device or sent via e-mail upon trial completion.

#### Signal processing and data assessment

All signal processing and data analysis was conducted in the MATLAB programming language. Real-time data display and processing on the tablet were implemented in Kotlin. Firmware was executed in embedded C. Signals were assessed in two ways. First, the periodicity was defined as the ratio of an autocorrelation peak for a subsequent beat to the primary peak.This periodicity is indicative of the repeatability of the signal and is a surrogate for signal-to-noise ratio. Second, the percentage vasoconstriction is calculated from the Hilbert analytic envelope, which tracks the amplitude of each PPG beat with time.

#### Calculation of autocorrelation for periodicity assessment

The autocorrelation for a given lag r(k) is a measurement of the correlation between the single variable time series waveform g(t) and g(t+k) where k=0,…,k is a random series. r(k) is thus defined as:

$$r\left(k\right)=\frac{c_{k}}{c_{0}}$$

where

$$c_{k}=\frac{1}{T}\sum_{t=1}^{T}\left(g\left(t\right)-{g\left(t\right)}^{*}\right)\left(g\left(t+k\right)-{g\left(t\right)}^{*}\right)$$

and $c_{0}$ is the sample variance of the waveform and T is the length of the time interval examined. Here, the periodicity is defined as the ratio of the 4 largest autocorrelation peaks to the peak at time zero.

#### Calculation of hilbert analytic envelope

Let g(t) represent the bio-signal waveform, H(t) the Hilbert transform, and ℵ(t) the analytic signal representation. ℵ(t) is composed of g(t) and H(t) as shown:

ℵ(t)=g(t)+iH(t)

H(t) can be defined using the Cauchy principal value (p.v.) by the convolution:

$$H\left(t\right)=\frac{1}{\pi }\int_{-\infty }^{\infty }\frac{g\left(\tau \right)}{t-\tau }d\tau$$

where τ is introduced as a temporary variable for the purposes of integration. The analytic envelope function is defined as the absolute value of the analytic function.

$$\left|ℵ\left(t\right)\right|=\sqrt{ℵ\left(t\right){ℵ\left(t\right)}^{*}}$$

where ${ℵ\left(t\right)}^{*}$ represents the complex conjugate of the analytic function.

In order to obtain the envelope in MATLAB, an ideal brick-wall sliding filter with a Kaiser window of length 500 (for a 50 Hz signal) and β of 8 is implemented. This filter w(n) is defined as:

$$w\left(n\right)=\frac{I_{o}\left(\beta \sqrt{1-{\left(\frac{2n}{N-1}-1\right)}^{2}}\right)}{I_{o}\left(\beta \right)}$$

where N is the length of the filter, n is incremented from 0 to N-1, and $I_{o}\left(\cdot \right)$ is the zeroth order modified Bessel function of the first kind

$$I_{o}\left(z\right)=\frac{1}{2\pi i}\oint e^{\left(\frac{z}{2}\right)\left(\frac{t+1}{t}\right)}t^{-1}dt$$

### Quantification and statistical analysis

All statistical details, including the study type, population size, and underlying distribution assumptions, for each study are provided in the figure legends and throughout the main text. For statistical analysis, MATLAB was used. Significance is defined at a P-value of 0.05. No collected data was excluded. Studies did not begin until the function of the device was verified.

## Data Availability

All data and code used in this study is available at https://github.gatech.edu/nzavanelli3/soft_sternal_patch/and additional assistance in analyzing the data will be provided upon reasonable request. The data presented in this study are summarized in the figures and available on request from the corresponding author.
