# Supplementary material for: Soft wireless sternal patch to detect systemic vasoconstriction using photoplethysmography
Source: iScience. 2023 Feb 13;26(3):106184. doi: 10.1016/j.isci.2023.106184 (PMC9985026; doi:10.1016/j.isci.2023.106184)
Supplement: Document S1. Figures S1–S9 and Tables S1–S3 [file mmc1.pdf]

iScience, Volume *26*

Supplemental information

Soft wireless sternal patch to detect systemic vasoconstriction using photoplethysmography

Nathan Zavanelli, Sung Hoon Lee, Matthew Guess, and Woon-Hong Yeo

Supplemental Information

Soft Wireless Sternal Patch to Detect Systemic Vasoconstriction using Photoplethysmography

Nathan Zavanelli[1,2], Sung Hoon Lee[2,3], Matthew Guess[1,2], and Woon-Hong Yeo[1,2,4,5,*]

[1] George W. Woodruff School of Mechanical Engineering, Georgia Institute of Technology, Atlanta, GA 30024, USA,
[2] IEN Center for Human-Centric Interfaces and Engineering at the Institute for Electronics and Nanotechnology, Georgia Institute of Technology, Atlanta, GA 30332, USA
[3] School of Electrical and Computer Engineering, Georgia Institute of Technology, Atlanta, GA 30332, USA
[4] Wallace H. Coulter Department of Biomedical Engineering, Georgia Tech and Emory University School of Medicine, Atlanta, GA 30332, USA
[5] Parker H. Petit Institute for Bioengineering and Biosciences, Neural Engineering Center, Institute for Materials, Institute for Robotics and Intelligent Machines, Georgia Institute of Technology, Atlanta, GA 30332, USA
***** Correspondence and Lead Contact: whyeo@gatech.edu; Tel.: +1-404-385-5710

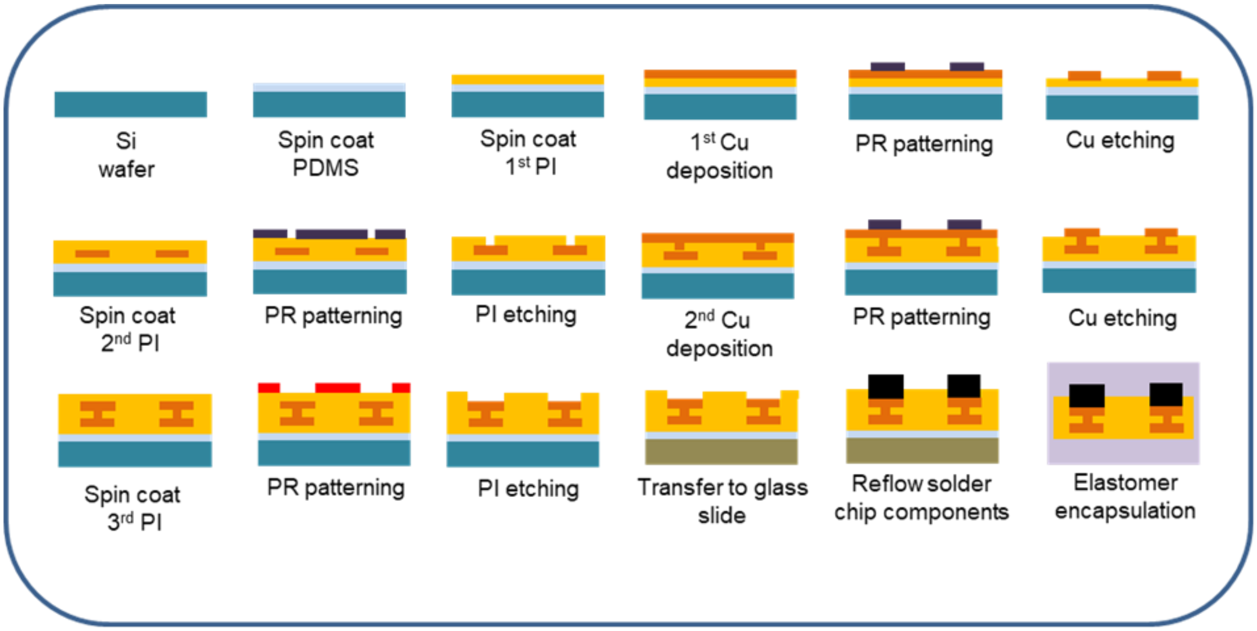

Supplemental Figure S1. Device microfabrication via photolithography and two-step transfer from an SI-coated wafer, related to STAR methods (device fabrication).

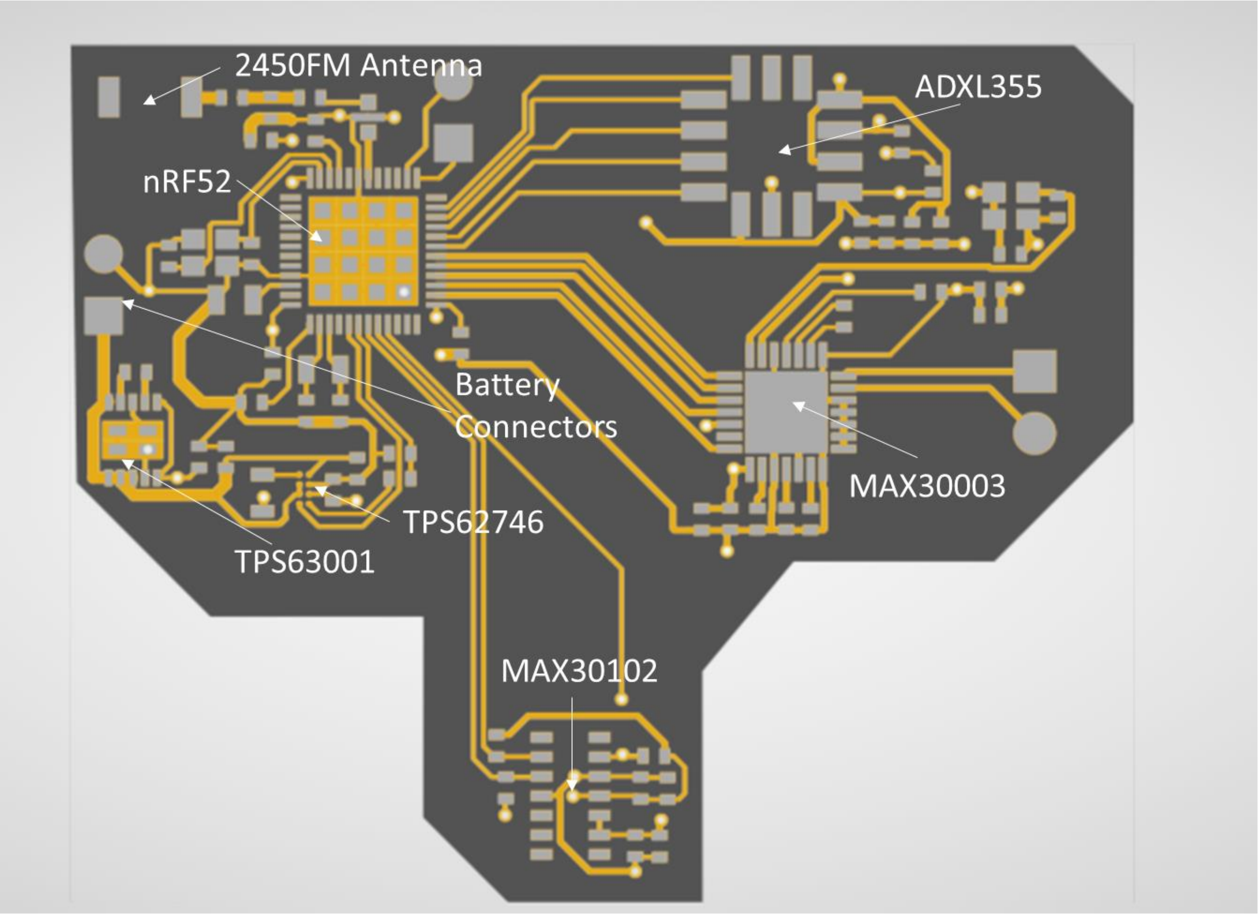

Supplemental Figure S2. Frontal illustration of the printed circuit board with annotations to mark the key components, related to STAR methods (circuit information).

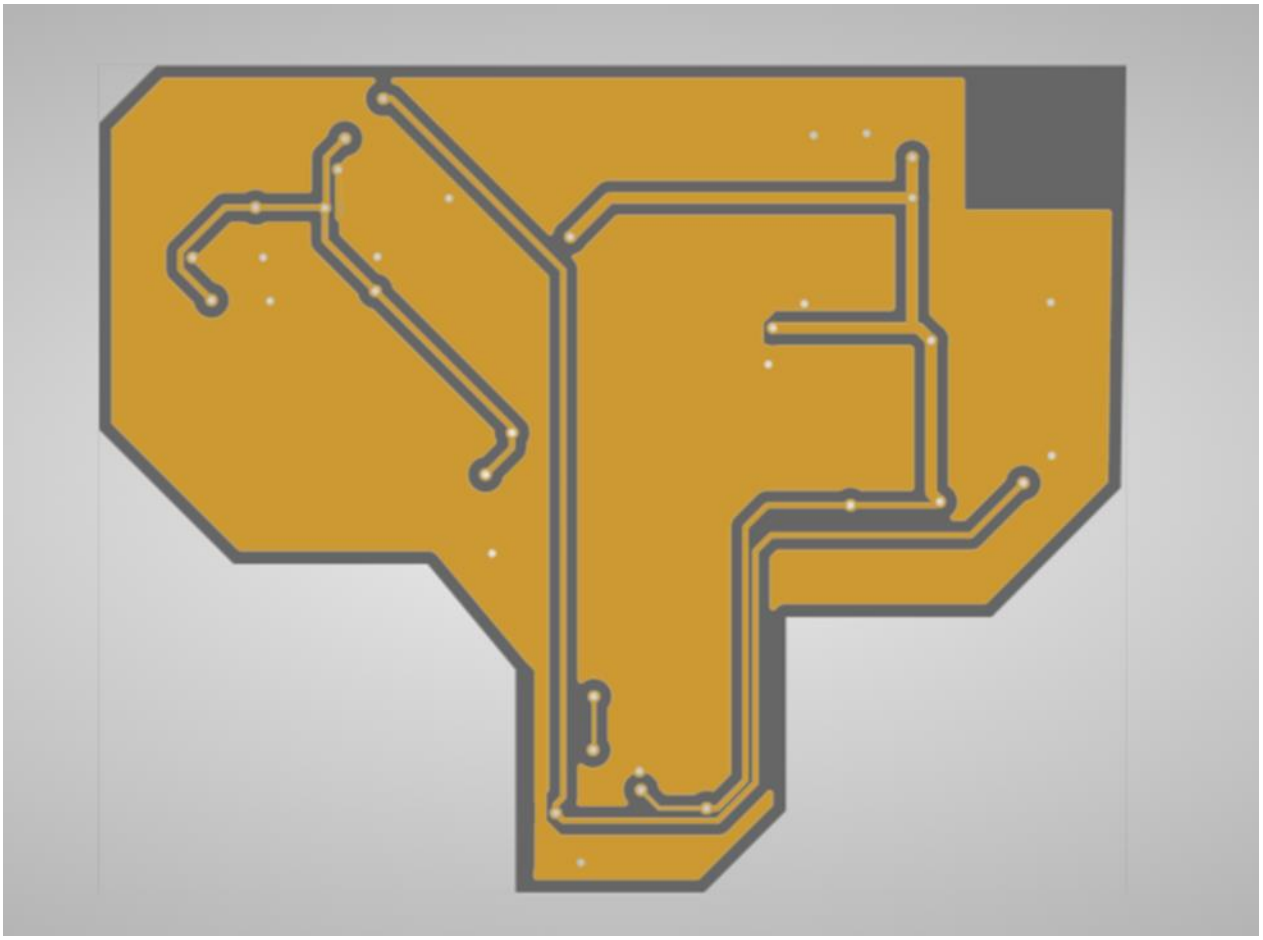

Supplemental Figure S3. Rear illustration of the printed circuit board, related to STAR methods (circuit information).

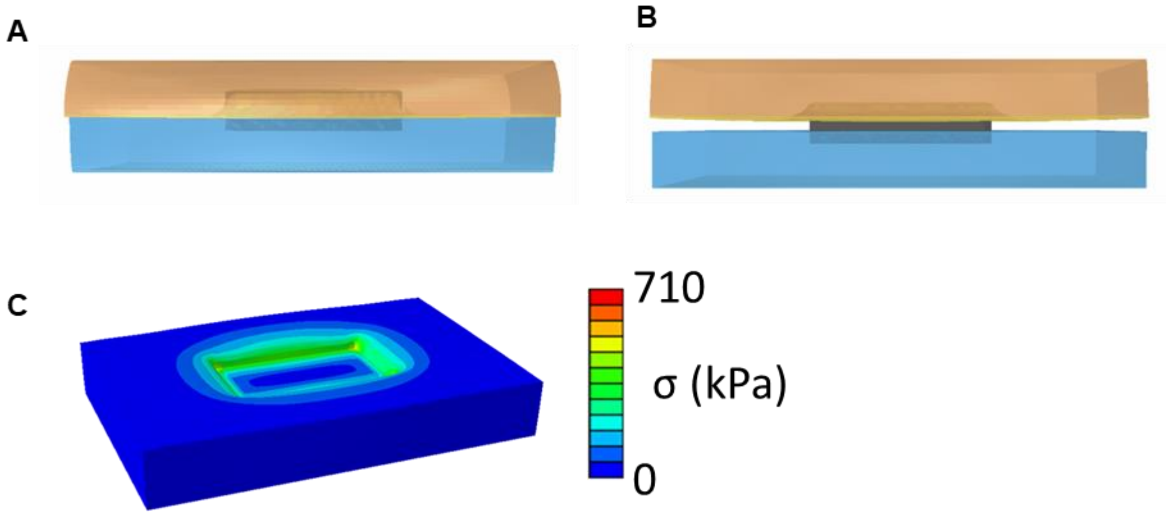

Supplemental Figure S4. FEA compression results, related to Figure 1. (A) FEA results for the soft device showing the elastomeric board encapsulating around the PPG unit and skin with an applied force of 1.1N. (B) Comparison FEA results for a 0.4mm flex PCB showing an inability to conform to the skin. (C) Simulation of stress applied to the user's skin.

A B

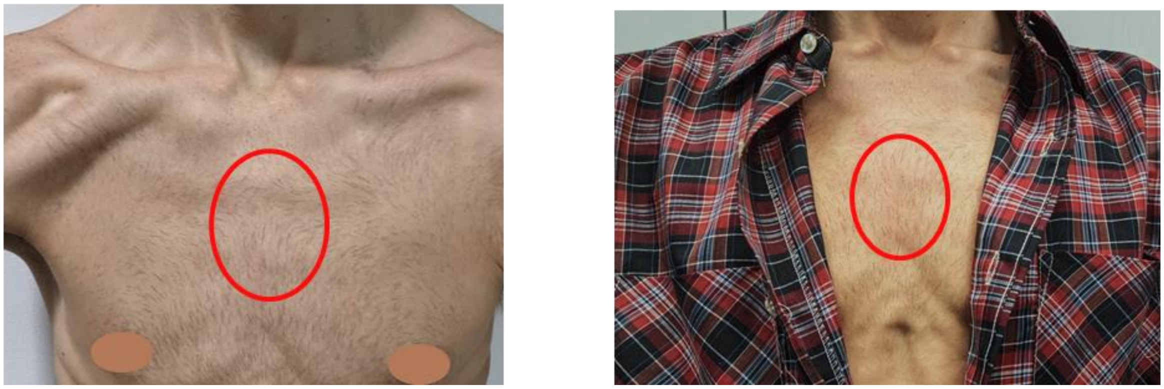

Supplemental Figure S5. Device biocompatibility assessment, related to Figure 2. (A) Image of the skin with the device area circled before device placement. (B) Image of the skin after 3 days of continuous wear, showing only a small red irritation in the circled device area.

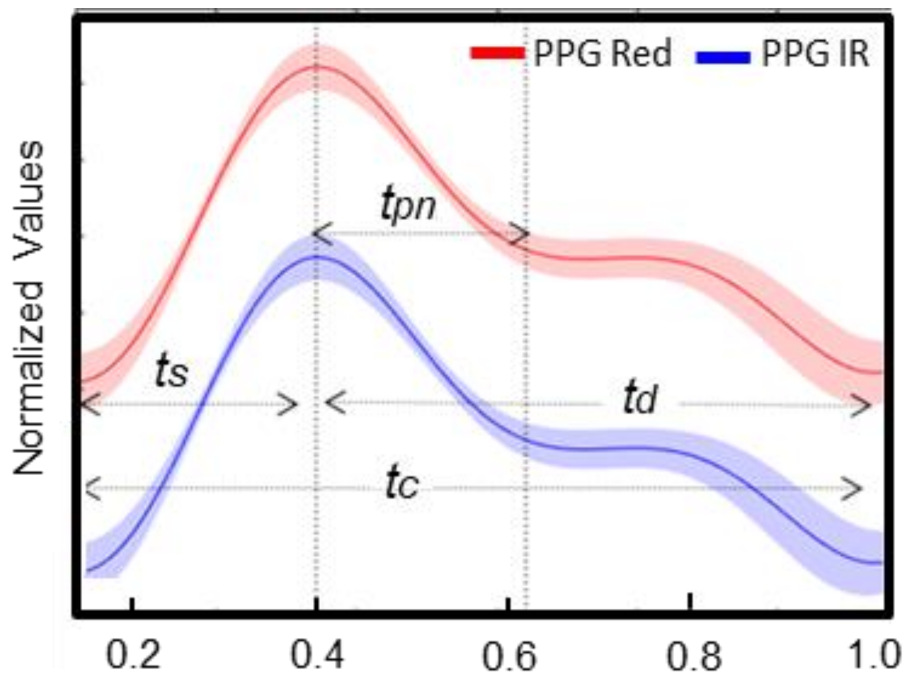

Supplemental Figure S6. Representative ensembled PPG signals with annotated fiducial marks, related to Figure 3.

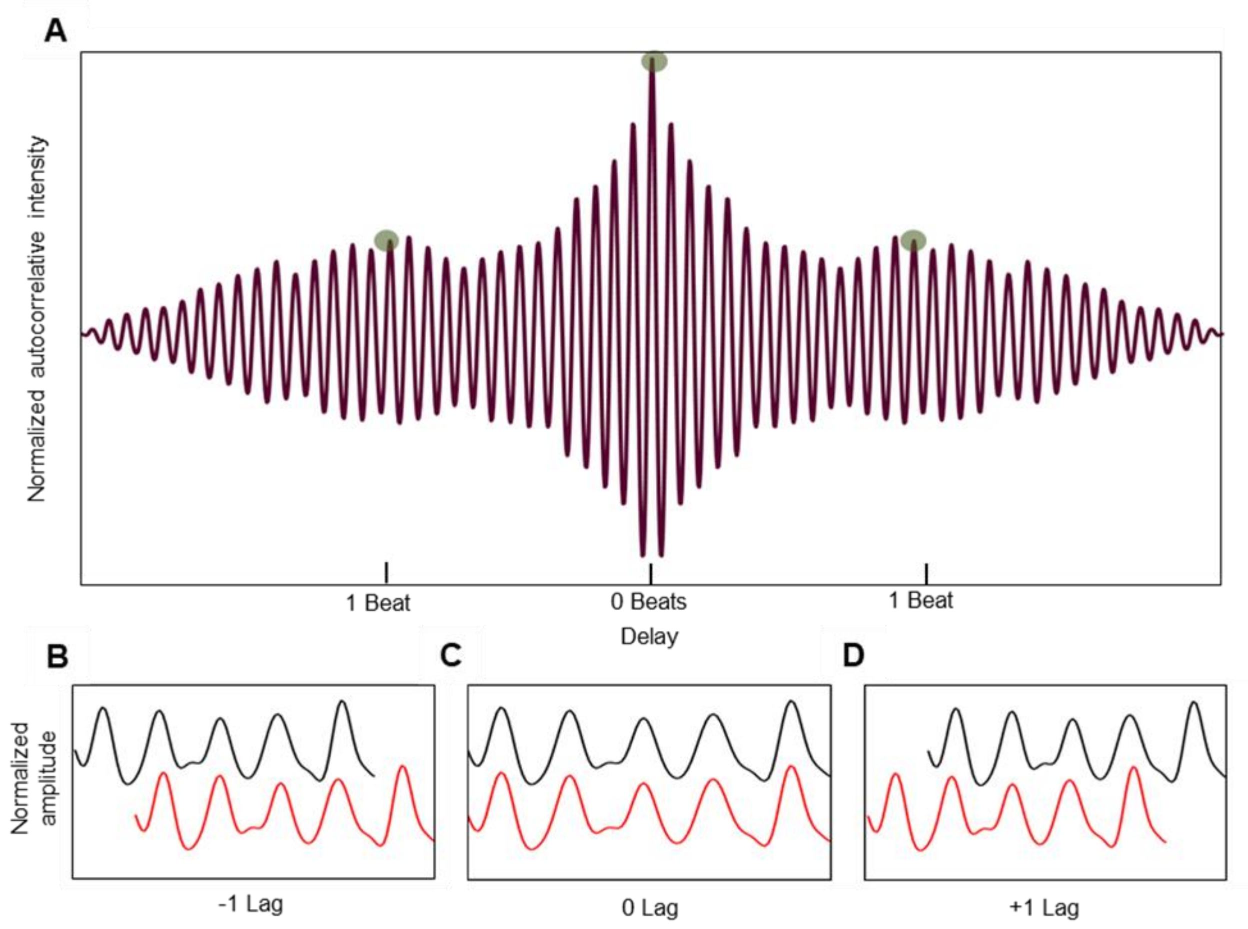

Supplemental Figure S7. Autocorrelation for the detection of PPG repeatability and signal quality, related to STAR methods (Calculation of autocorrelation for periodicity assessment). (A) Plot of the PPG autocorrelation defined at the 1 and 0 beat lags. The ratio of the successive lag peaks to the fundamental peak is the autocorrelative periodicity. (b-d) Time series PPG signals plotted for -1 (B), 0 (C), and 1(D) beat lags.

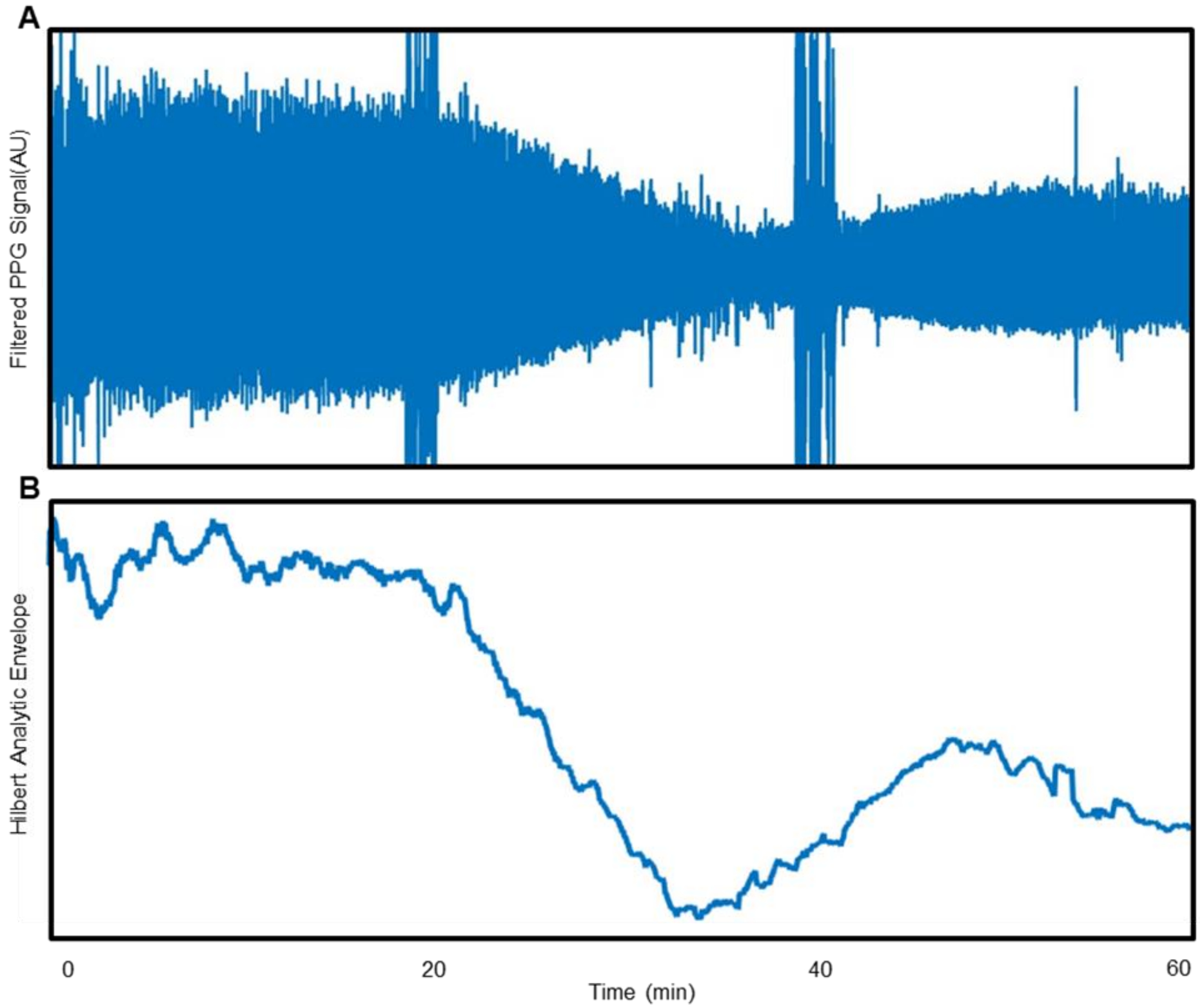

Supplemental Figure S8. Demonstration of the Hilbert analytical envelope in a representative vasoconstriction, related to STAR methods (Calculation of Hilbert analytic envelope). (A) the filtered signal and (B) the corresponding Hilbert envelope, which represents the amplitude of each PPG wave.

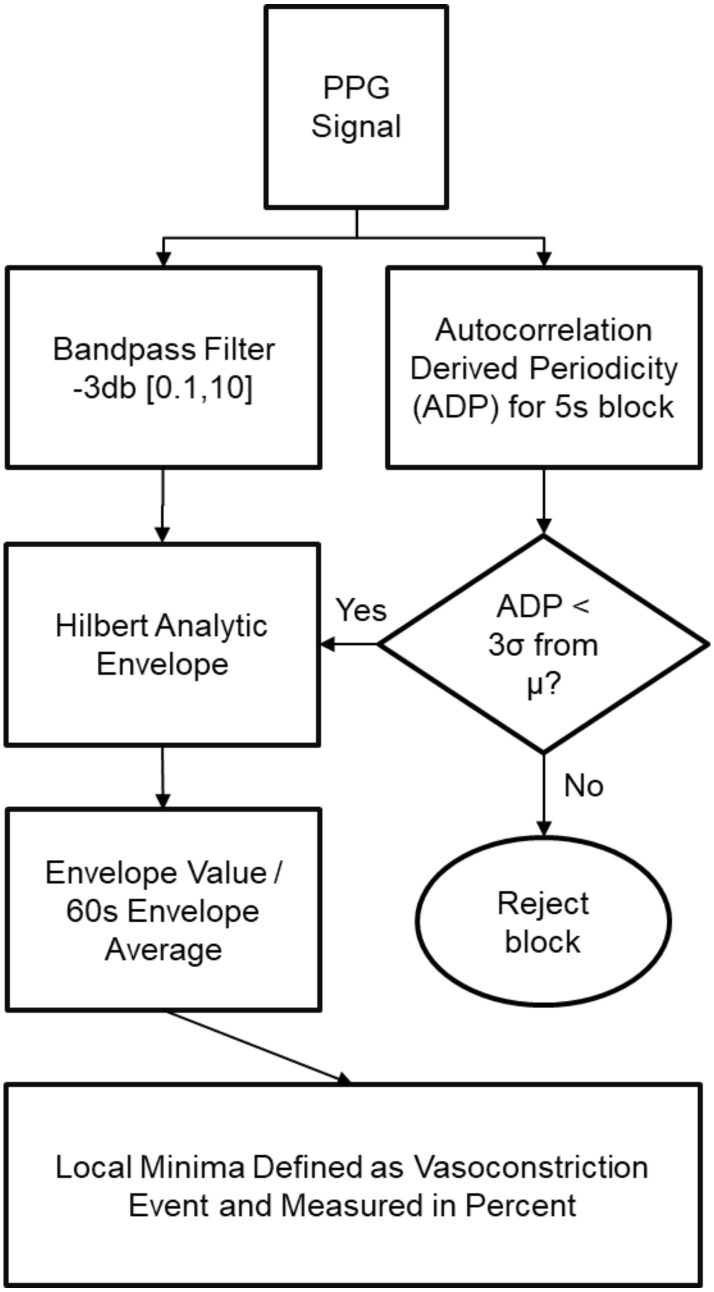

Supplemental Figure S9. Flow diagram of signal processing for percent vasoconstriction detection, related to STAR methods (Signal processing and data assessment).

Supplemental Table S1: Summary of human subjects who volunteered for the location optimization study, related to STAR methods (Experimental Model and Subject Details).

Subject	Age	BMI	Gender	Race
1	24	19	M	White
2	50	29	F	White
3	28	28	M	African American
8	28	24	M	Middle-Eastern
5	51	40	M	White

Supplemental Table S2: Summary of human subjects who volunteered for the vasoconstriction validation study, related to STAR methods (Experimental Model and Subject Details).

Subject	Age	BMI	Gender	Race
1	24	19	M	White
2	32	24	M	White
3	50	29	F	White
4	28	28	M	African American
5	35	24	M	Asian
6	22	26	M	White
7	20	25	M	Middle-Eastern
8	28	24	M	Middle-Eastern
9	51	40	M	White

Supplemental Table S3: Summary of human subjects who volunteered for the overnight apnea comparison study, related to STAR methods (Experimental Model and Subject Details).

Subject	Age	BMI	Gender	Race	Stop-Bang/ PSQI/ ESS/ ISI	OSA
1	31	23.2	M	White	Low/9/0/9	Mild (5<x<15)
2	35	29.3	M	White	Low/7/9/7	Moderate (15<x<30)
3	59	35.2	M	White	High/7/4/3	Moderate-Severe (25<x<40)
4	35	27.6	M	Asian	Low/6/3/7	Mild (5<x<15)
5	51	39.4	M	White	Medium/6/4/7	Moderate (15<x<30)
